# Effects of Zinc Pollution and Compost Amendment on the Root Microbiome of a Metal Tolerant Poplar Clone

**DOI:** 10.3389/fmicb.2020.01677

**Published:** 2020-07-15

**Authors:** Francesco Guarino, Giovanni Improta, Maria Triassi, Angela Cicatelli, Stefano Castiglione

**Affiliations:** ^1^Department of Chemistry and Biology “A. Zambelli”, University of Salerno, Salerno, Italy; ^2^Department of Public Health, University of Naples Federico II, Naples, Italy

**Keywords:** phytoremediation, metals, compost, microbiome, bacteria, fungi, NGS

## Abstract

Until recently, many phytoremediation studies were focused solely on a plants ability to reclaim heavy metal (HM) polluted soil through a range of different processes, such as phytoextraction and phytostabilization. However, the interaction between plants and their own rhizosphere microbiome represents a new research frontier for phytoremediation. Our hypothesis is that rhizomicrobiome might play a key role in plant wellness and in the response to external stimuli; therefore, this study aimed to shed light the rhizomicrobiome dynamics after an organic amendment (e.g., compost) and/or HM pollution (e.g., Zn), and its relation with plant reclamation ability. To reach this goal we set up a greenhouse experiment cultivating in pot an elite black poplar clone (N12) selected in the past for its excellent ability to reclaim heavy metals. N12 saplings were grown on a soil amended with compost and/or spiked with high Zn doses. At the end of the experiment, we observed that the compost amendment strongly increased the foliar size but did not affect significantly the Zn accumulation in plant. Furthermore, the rhizomicrobiome communities (bacteria and fungi), investigated through NGS, highlighted how α diversity increased in all treatments compared to the untreated N12 saplings. Soil compost amendment, as well as Zn pollution, strongly modified the bacterial rhizomicrobiome structure. Conversely, the variation of the fungal rhizomicrobiome was only marginally affected by soil Zn addition, and only partially impaired by compost. Nevertheless, substantial alterations of the fungal community were due to both compost and Zn. Together, our experimental results revealed that organic amendment increased the bacterial resistance to external stimuli whilst, in the case of fungi, the amendment made the fungi microbiome more susceptible. Finally, the greater microbiome biodiversity does not imply, in this case, a better plant wellness or phytoremediation ability, although the microbiome plays a role in the external stimuli response supporting plant life.

## Introduction

Heavy metal (HM) contamination of the environment has increased to levels that are harmful for living organisms, mainly because of anthropogenic activities. HMs are non-degradable pollutants, and, thus, they persist indefinitely in diverse environmental matrices. Among HMs, zinc (Zn) can be included; it has an atomic number of 30 and atomic weight of 65.38, it is the 24rd most abundant element on earth, and it is an essential trace element for all living beings, including plants. Zinc is a constituent of many proteins, it is also an enzyme cofactor and it is fundamental for optimum plant growth and development (Broadley et al., 2007). However, at high concentrations in the soil, Zn is phytotoxic, and plants that accumulate it through root absorption or deposition, pose health risks to consumers (Bolan et al., 2014). Therefore, remediation of HM polluted soils is imperative and necessary to reduce their impact on plants, ecosystems, landscape, soil microbial biodiversity and human health.

Phytoremediation is a green bio-technology, solar driven and cost-effective, associated with many additional benefits such as: conversion of plant biomass into bioenergy, sustaining of biodiversity, soil stabilization, and numerous other ecosystem services. Over the past decade, it has become clear that phytoremediation is assisted by soil or rhizosphere microorganisms often useful, and sometime necessary, to increase HM bioavailability and their subsequent uptake by plants not clear (Becerra-Castro et al., 2013; Ahemad and Kibret, 2014; Kamran et al., 2017). Recent evidence emphasizes that the success of phytoremediation of HM contaminated environments depends strongly on plant-microbiome interactions (Cicatelli et al., 2019). Plants and microbes coexist or compete for survival and their interactions play a vital role in plant adaption to HM pollution. Moreover, microorganisms, and their interaction with HMs in contaminated soils can contribute to their removal and, therefore, influence the efficiency and rate of the phytoremediation. Rhizosphere microorganisms, especially bacteria and fungi, colonize plant roots (Ahemad and Kibret, 2014), establishing an extensive microbial network that is mutually beneficial. The host plant constantly nourishes the microbiota through root exudates (up to 30% of photosynthesis by products) (Vives-Peris et al., 2020). The presence of the root apparatus, in fact, is spread throughout the microbial network, that perceives it and responses in turn, also affecting mobility and bioavailability of the HMs in the soil and in the rhizosphere, thereby protecting the plant from their toxicity (Shafigh et al., 2019). The amount and composition (simple molecules, sugars, organic acids, and secondary metabolites, as well as complex polymers, such as mucilage) of exudates vary in relation to the host genotype, developmental stage and, moreover, they are modulated by abiotic stresses (Rengel, 2015). In turn, rhizobacteria can promote plant growth by secreting beneficial chemical compounds, such as mineral phosphate solubilizers, indole acetic acid (IAA), siderophores, and ACC deaminase (Wang et al., 2018; Gupta and Pandey, 2019), affecting the absorption of pollutants by changing soil pH, excreting surfactants or chelating substances (Rajkumar et al., 2012) and altering redox potentials (Rengel, 2015). Rhizosphere fungi, such as mycorrhiza, are also able to enlarge soil exploration of plant roots, improving the uptake and translocation of nutrients and HMs from soils to the different plant organs. Currently, phytoremediation has not yet reached the level of highly efficient and fast clean-up technologies, and therefore many studies have focused their attention on strategies to improve soil phytoremediation efficiency, also using microbes able to assist plants in the processes increasing HM bio-availability and accessibility, plant growth, etc. A promising strategy for the managing of HM contaminated soils includes the use of metal tolerant poplar clones. In fact, it is widely recognized that the genus *Populus* has several features that are suitable for effective soil reclamation, such as rapid growth, a deep and spreading root system, marked adaptability to different pedoclimatic conditions and remarkable capability to vegetative reproduction, which makes its propagation quite easy. In addition, poplars are highly tolerant of different contaminants, and a large clonal variability in metal-resistance or accumulation traits (Kopponen et al., 2001; Laureysens et al., 2005; Dos Santos Utmazian and Wenzel, 2007; Cicatelli et al., 2014). Several poplar clones, belonging to the species *Populus alba* L. and *Populus nigra* L., have been screened for their HM tolerance during a field trial on a soil highly polluted by Cu and Zn. Among these, a black poplar clone named N12 was selected for its high survival, HM tolerance and accumulation (Castiglione et al., 2009).

In the present study, Zn phytoremediation, operated by the N12 black poplar clone, was assisted by compost (CMP) soil amendment. Compost may be easily obtained from artificially controlled microbial degradation of organic wastes [e.g., municipal solid and agricultural organic wastes (Singh and Agrawal, 2008)]. Compost can be used to improve the physico-chemical and biological properties of the soil, by modifying its porous structure (for an improved root penetration), water storage capacity and resistance to erosion, and also by introducing new organic matter, nutrients and microbes. All of these contribute to increased crop growth and yield, and provide additional genera and species of microorganisms potentially useful to plant health (Sudharsan Varma and Kalamdhad, 2015).

Several studies have clarified the structure and variation of root-associated microbiomes in different plants species employed in bio- phytoremediation (Jamir et al., 2019; Zadel et al., 2020). At present, as far as we know, very little information is available on how microbial communities assemble in the rhizosphere, defined as the top soil layer of 1–2 mm surrounding the plant roots, if it is polluted by a metal such as Zn and amended with compost (CMP). The main objectives of our study were to: (i) investigate the Zn phytoremediation performance of the N12 multi-metal tolerant black poplar clone, assisted by CMP amendment, and (ii) investigate the changes of its associated rhizosphere microbiome in relation to Zn addition to the soil and CMP amendment.

## Materials and Methods

### Experimental Design

The experiment was conducted using N12 poplar cuttings (12) sprouting separately in single pots and grown in a greenhouse to minimize the impact of other environmental stress factors. Pots were filled using an agricultural soil (Supplementary Table S1, T0 soil). One portion of this soil was amended with certified high-quality compost (CMP, 20% of the pot volume) obtained from the organic fraction of municipal solid waste. The N12 poplar clone, belonging to a collection of *Populus nigra* L. that originated from an Italian natural population, was selected for this experimentation because during a field trial on a multi-metal polluted site (Castiglione et al., 2009) it was shown to be tolerant to high concentrations of Cu and Zn. Cuttings (20 cm long) were placed overnight under running tap water and then singularly put into plastic pots of 5 liters of volume (3 cuttings per treatment group), containing soil amended or not with CMP. After 2 months of growth, Zn was added to the soil as a nitrate salt, avoiding the solution leaching, in three successive doses every week, up to a final concentration of 450 mg kg^–1^ soil dry weight (DW). The pot trial included the following four experimental groups: CNT (3 plants on unpolluted soil); Zn450 (3 plants on Zn polluted soil); CMP (3 plants on unpolluted and CMP amended soil); CMP + Zn450 (3 plants on Zn polluted and CMP amended soil).

### Leaf Morphometric Measurement

At the end of experimentation, leaf area and length, average and maximum width were recorded on five completely expanded leaves of each poplar plant, using a portable leaf area meter (LI-3000C; LI-COR, Nebraska, United States). The total number of measured leaves is 60.

### Plant and Soil Collection

At the end of the experimentation (about 3 months after planting), whole intact saplings were harvested from each pot. Roots, carefully washed with distilled water to eliminate soil sediments, stems and leaves were collected and separately dried at 75°C to constant weight, for measurements of their DW and for estimation of Zn content. At the start of the experimentation, soils, CMP amended or not, were analyzed to evaluate the main physical and chemical features. For pH determination, 10 g of soil were placed in 25 mL of deionized water and shaken for 2 h. Soil organic carbon and Organic Matter Content (OMC) were determined using the Walkley and Black (1934) protocol. For determination of available phosphate, the method described by Olsen et al. (1954) was used. Total C, total N, and the C/N ratio were determined through the combustion method of elemental analysis. The barium chloride triethanolamine method (Youden and Mehlich, 1938) was employed to estimate soil cation exchange capacity (CEC). Furthermore, three soil cores were collected at the start and at the end of experimentation from pots of each experimental group to determine the total and available Zn metal content. Soil cores were pooled, mixed, sieved (2 mm) and dried at 75°C to constant weight and processed for metal content analysis as described below.

### Plant and Soil Metal Content

Dried soils were pulverized in a planetary ball mill (PM4, Retsch, Germany), while plant organs were pulverized in a mortar (leaves, roots) using liquid nitrogen, or reduced to ash (stems) by baking at 550°C for 2 h. For each plant organ, three biological replicates were analyzed. All the matrices (soils and plant organs) were digested with an acid mixture (HNO_3_ 65%; HF, 50% = 2:1v/v) in a microwave oven (Milestone Srl – 24010, Sorisole BG, Italy) using the following digestion program: 1 min at 250 W, 1 min at 0 W, 5 min at 250 W, 4 min at 400 W, 4 min at 600 W, 5 min at 250 W. The method of Lindsay and Norvell (1978) was used to estimate available Zn concentrations in soil extracts obtained from the dried granulometric fraction. Element concentrations were determined by ICP-OES (Optima 7000DV. PerkinElmer Italia Spa – 20126, Milan, Italy). Standard reference material (Mackey et al., 2004) was analyzed in order to verify the accuracy of the obtained results. Standard solutions of Zn were used to generate the calibration curve to convert emission readings into the analyte concentrations.

### Rhizosphere Soil Collection and DNA Extraction

At the end of experimentation, soil particles adhering to the fine roots, with a diameter of about 2 mm or less, were sampled and considered as rhizosphere soil, while the fine roots were collected from poplar plants of each experimental group, pooled and placed in a tube containing 25 mL of sterile physiological solution (0.9% NaCl). Tubes were vortexed at maximum speed for 15 s and then shaken for 1 h at room temperature to release the majority of the microorganisms adhering to the roots. Roots were recovered and transferred to a new sterile 50-mL tube with 25 mL of physiological saline (0.9% NaCl), sonicated at low frequency for 5 min to further disrupt tiny soil aggregates and recover the attached microbes. The roots were then removed from this solution. The solutions containing fine sediment and microorganisms were pooled and centrifuged for 20 min at 5000 rpm to pellet the microorganisms. Pelleted microorganisms were washed twice with physiological saline, and finally resuspended in 20 mL of the same solution. Aliquots of 1 ml were flash-frozen in liquid nitrogen and stored at −80°C until processing. Aliquots of rhizosphere solutions (100 μL) were mixed in the lysis buffer of the DNA EXTRACT-N-AMP kit (Sigma-Aldrich, Milan, Italy), following the supplier instructions, for a rapid DNA extraction.

### Amplicon Library Preparation and NGS Sequencing

Microbial DNAs were extracted in triplicate from rhizosphere solution of each experimental group and PCR amplified. The V3-V4 region of the bacterial 16S rRNA genes was amplified with 341F (5′-CCTACGGGRSGCAGCAG-3′) and 909R (5′-TTTCAGYCTTGCGRCCGTAC-3′) specific primers, the following PCR thermal profile was used: initial denaturation at 95°C for 3 min, followed by 40 cycles of denaturation at 95°C for 1 min, annealing at 58°C for 1 min and elongation at 72°C for 1 min. with additional final elongation step at 72°C for 5 min. The fungi ITS2 region was amplified using the ITS3 (5′-GCATCGATGAAGAACGCAGC) and ITS4 (5′-TCCTCCGCTTATTGATATGC-3′) primers and the following PCR thermal profile was employed: initial denaturation at 95°C for 3 min, followed by 40 cycles of denaturation at 95°C for 1 min, annealing at 55°C for 1 min and elongation at 72°C for 1 min, with additional final elongation step at 72°C for 5 min. The amplicon libraries were processed following the manufacturer’s instructions (BMR Genomics, Padua, Italy) and sequenced by BMR Genomics (Padua, Italy).

### Sequences Analysis

Illumina sequence data were sorted based on unique barcodes and quality-controlled using the Quantitative Insights Into Microbial Ecology (Qiime2, version 2017.8)^1^ with plugins-demux^2^ dada2 (Callahan et al., 2015) and feature-table (McDonald et al., 2012). α - and β-diversity analyses were performed by using plugins alignment (Katoh and Standley, 2013) diversity^3^. For taxonomic analysis, a pre-trained Naive Bayes classifier based on the SILVA 138 (Operational Taxonomic Units) OTUs database, in the case of 16S rDNA^4^, which has been trimmed to include the V3-V4 region of 16S rRNA gene, bound by the 341F/909R primer pair, was used. While, the classifier, for fungi ITS2 DNA sequences, was pre-trained on UNITE database version 7–99%, and applied to paired-end sequence reads to generate taxonomy tables. Taxonomic and compositional analyses were conducted by using plugins feature-classifier^5^ (Bokulich et al., 2018), taxa^6^ and composition (Mandal et al., 2015).

### Microbiome Diversity Indices

The raw data were used to prior α-diversity analyses using Observed OTUs Shannon Simpson and Chao1 metrics in Qiime2 α-diversity plugins (Faith, 1992). The total frequency that each sample was be rarefied to prior to computing diversity metrics was 6820 in the case of 16S rDNA and 59788 in the case of ITS. The differences among the experimental groups were assessed with the Kruskal-Wallis test. β-diversity was estimated by calculating the Dice non-phylogenetic β-diversity distance (Lozupone and Knight, 2005). All values were expressed as means ± standard deviations of triplicate analyses. Analysis of variance (PERANOVA) was performed and *P*-values were then obtained using 999 permutations. Linear Discriminant Analysis (LDA) Effect Size (LEfSe) was used to detect the bacterial taxonomic biomarkers across the different treatments (Segata et al., 2011).

### Statistical Analysis

A preliminary test to assay the Gaussian distribution, homogeneity variance and homoscedasticity were performed on data in R environment (R Development Core Team, 2011) through Shapiro-Wilk test, Levene test and Bartlett test, respectively (Shapiro and Wilk, 1965). After that, biomass production, morphometric data, metal concentration and accumulation in different plant organs and soil were tested in R environment by Kruskal and Wallis One-Way Analysis of Variance by ranks (Wallis, 2008), followed by *post hoc* Nemenyi test (Graves et al., 2015).

## Results

### N12 Sapling Growth

**FIGURE 1:**
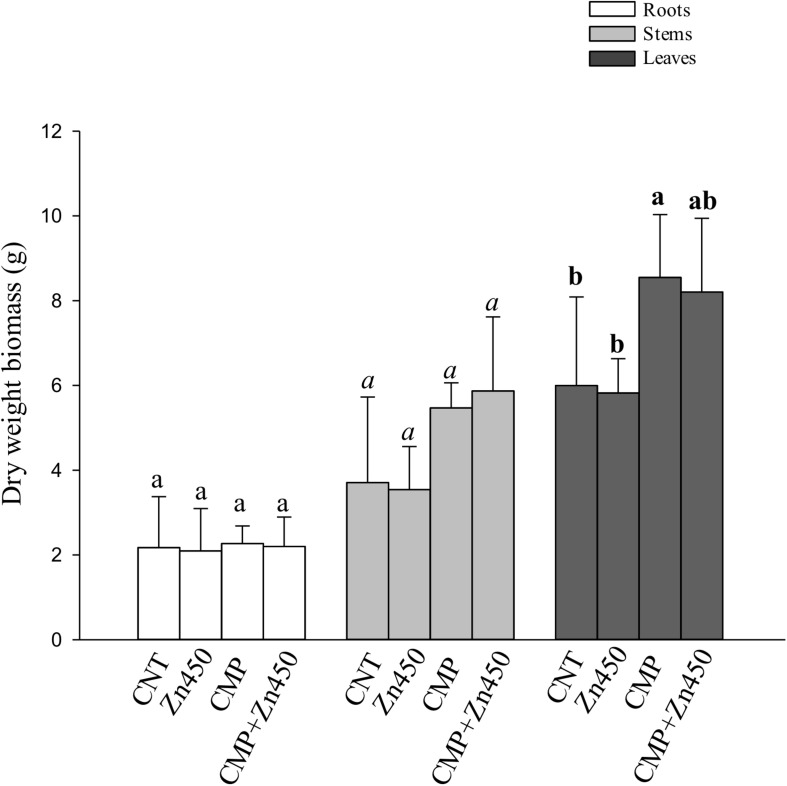
Biomass (dry weight, g) of N12 leaves, roots and stems at harvest and for each treatment. Different letters indicate statistically significant differences among treatments compared to the leaves (*p* < 0.05). Bars indicate standard deviations.

At the end of experimentation, N12 poplar saplings, grown on Zn spiked soils, did not show any symptoms of toxicity or stress. Zinc addition had no effect on biomass production of roots, stems and leaves (Figure 1). In contrast, the 20% CMP soil amendment improved growth and exerted a positive effect on the poplar saplings, mainly on their leaves (Table 1). Specifically, leaf biomass was significantly greater in saplings grown on CMP amended soil (without Zn) if compared with those grown on not-amended soils (CNT and Zn450). Moreover, poplar saplings belonging to CMP group had very large and dark green leaves. The morphometric analysis revealed that the leaves of saplings grown on CMP amended soils were significantly more expanded than those of saplings grown on unamended ones (Table 1).

**TABLE 1:** Leaf area (cm^2^), length, average width and maximum width (cm) of N12, grown under different conditions, were determined (mean value ± standard deviation five replicates for each sapling of each experimental thesis).

	**Leaf area**	**Leaf length**	**Average width**	**Maximum width**
CNT	27.05 ± 9.18 ^a^	6.92 ± 1.20^a^	3.83 ± 0.87^a^	6.15 ± 1.07^a^
Zn450	19.59 ± 3.35^a^	5.91 ± 1.02^a^	3.23 ± 0.30^a^	5.29 ± 0.53^a^
CMP	42.81 ± 8.02^b^	9.16 ± 1.54^b^	4.61 ± 0.51^ab^	8.07 ± 0.70^b^
CMP + Zn450	48.22 ± 9.53^b^	9.77 ± 1.41^b^	4.90 ± 0.82^b^	8.62 ± 1.03^b^

*Different letters indicate significantly different (*P* < 0.05) values for treatments with reference to the same leaf morphometric parameter.*

### Soil Characterization

Physico-chemical analyses were performed on unplanted soils with and without CMP amended, and both soils showed similar results (Supplementary Table S1). In unplanted soils, the total amount of Zn was below the guideline values established for a green area, and the bioavailable fractions of Zn were low. At the end of the experiment, Zn concentrations increased, as a consequence of the artificial contamination of both soils (data not shown). The available fractions of Zn, initially low, were not significantly modified in the presence of poplar saplings as well as by CMP soil amendment (data not shown).

### Zn Content in N12 Saplings

**FIGURE 2:**
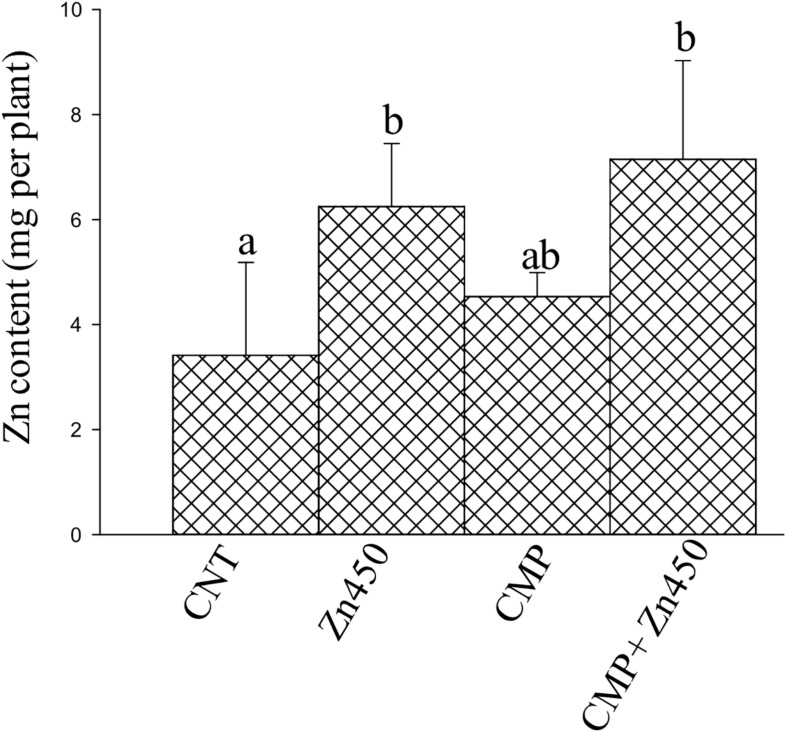
Total Zn amount in the N12 saplings at the end of experiment and for each treatment. Different letters indicate significantly different values for treatments (*p* < 0.05). Bars indicate standard deviations.

Zinc reached the highest concentrations in the roots and stems (ca. 1,200 and 800 μg g^–1^, respectively) and the lowest in leaves (ca. 190 μg g^–1^; Table 2) in Zn450 and CMP + Zn450 treatments. The total amount of Zn accumulated by the total biomass of the saplings increased after Zn addition, but with no significative difference between the two experimental groups with CMP soil amendment (Figure 2).

**TABLE 2:** Zinc concentrations (μg g^–1^) in roots, stems and leaves of the N12 grown under different conditions (mean value ± standard deviation of three replicates for each experimental thesis).

	**Roots**	**Stems**	**Leaves**
CNT	346.82 ± 44.38^a^	596.98 ± 18.88	71.78 ± 2.41
Zn450	1247.77 ± 525.62^b^	800.60 ± 59.40	194.07 ± 5.47
CMP	261.58 ± 8.68^a^	626.82 ± 19.45	73.15 ± 25.49
CMP + Zn450	680.75 ± 84.20^c^	817.81 ± 24.34	87.91 ± 18.93

*Different letters indicate significantly different (*P* < 0.05) values for treatments with reference to the same organ.*

### The Rhizosphere Microbiome of N12 Saplings

The rhizosphere microbiome of N12 poplar saplings grown on a soil spiked with Zn with and without CMP amendment, was analyzed. In addition, phytoremediation capability of the saplings was investigated in relation to rhizosphere microbiome biodiversity. In order to evaluate the interaction between microorganisms living on the root surface and the saplings, and more specifically, to investigate a correlation between phytoremediation capacity of this clone and its rhizosphere microbiome, DNAs were isolated from microbial communities collected from fine roots surface and the 16S rDNA and ITS regions were PCR amplified and deep sequenced.

#### Biodiversity of the Root Bacterial Microbiome

For 16S rDNA, 160,000 gene from 12 samples were sequenced with an average read length of 600 bp (300 × 2). Quality filtering, denoising and the removal of chimeric sequences reduced this number to 108,387. The ASV was 436 and the rarefaction curves are reported in Supplementary Material (Supplementary Figure S1). The α biodiversity was estimated, the Observed OTUs and both Shannon and Simpson indices calculated. The Kruskal-Wallis pairwise comparison among the four treatments (CNT, Zn450, CMP, CMP + Zn450) were not statistically significant for Observed OTUs, whilst all treatments affected both Shannon and Simpson indices (*p* < 0.05) when compared to CNT (Table 3).

**TABLE 3:** Kruskal-Wallis pairwise comparison of two α-diversity indices **(A)** Shannon and **(B)** Simpson among the experimental groups.

**Thesis 1**	**Thesis 2**	**H**	***p*-value**	***q*-value**
**(A) Shannon index**				
**Thesis 1**	**Thesis 2**	**H**	***p*-value**	***q*-value**

**CNT (*n* = 3)**	**CMP (*n* = 3)**	**3.86**	**0.049**	**0.099**
**CNT (*n* = 3)**	**CMP + Zn450 (*n* = 3)**	**3.86**	**0.049**	**0.099**
**CNT (*n* = 3)**	**Zn450 (*n* = 3)**	**3.86**	**0.049**	**0.099**
CMP (*n* = 3)	CMP + Zn450 (*n* = 3)	0.43	0.512	0.615
CMP (*n* = 3)	Zn450 (*n* = 3)	0.05	0.827	0.827
CMP + Zn450 (*n* = 3)	Zn450 (*n* = 3)	0.43	0.512	0.615

**(B) Simpson index**				
**Thesis 1**	**Thesis 2**	**H**	***p*-value**	***q*-value**

**CNT (*n* = 3)**	**CMP (*n* = 3)**	**3.86**	**0.049**	**0.099**
**CNT (*n* = 3)**	**CMP + Zn450 (*n* = 3)**	**3.86**	**0.049**	**0.099**
**CNT (*n* = 3)**	**Zn450 (*n* = 3)**	**3.86**	**0.049**	**0.099**
CMP (*n* = 3)	CMP + Zn450 (*n* = 3)	1.19	0.275	0.275
CMP (*n* = 3)	Zn450 (*n* = 3)	1.19	0.275	0.275
CMP + Zn450 (*n* = 3)	Zn450 (*n* = 3)	1.19	0.275	0.275

*Statistically significant values are indicated in bold (*p* < 0.05).*

The α diversity was greater in the case of CMP, CMP + Zn450 and Zn450 compared to CNT (*p* < 0.05). Correlation analyses revealed that Observed OTUs were negatively correlated with total sapling biomass (*r* = −0.57, *p* < 0.05) and total sapling Zn accumulation (*r* = −0.58, *p* < 0.05). In particular, the number of Observed OTUs was negatively correlated with root (*r* = −0.58, *p* < 0.05) and stem biomass (*r* = −0.60, *p* < 0.05), and stem Zn accumulation (*r* = −0.58, *p* < 0.05).

The β diversity was calculated in order to highlight the differences among the experimental groups using the Dice metrics. The results (Table 4) showed that the Dice distance among the analyzed groups was statistically significative (*p* ≤ 0.1).

**TABLE 4:** The pairwise PERMANOVA tests whether Dice distances between samples within the same experimental group are more similar to each other than they are to samples from the other groups.

**Thesis 1**	**Thesis 2**	**Sample size**	**Permutations**	**pseudo-F**	***p*-value**	***q*-value**
**CNT**	**CMP**	**6**	**999**	**6.19**	**0.090**	**0.110**
**CNT**	**CMP + Zn450**	**6**	**999**	**7.57**	**0.100**	**0.110**
**CNT**	**Zn450**	**6**	**999**	**5.71**	**0.100**	**0.110**
**CMP**	**CMP + Zn450**	**6**	**999**	**4.95**	**0.090**	**0.110**
**CMP**	**Zn450**	**6**	**999**	**7.20**	**0.100**	**0.110**
**CMP + Zn450**	**Zn450**	**6**	**999**	**8.52**	**0.100**	**0.110**

*Statistically significant values are indicated in bold (*p* ≤ 0.1).*

##### Bacterial community composition

**FIGURE 3:**
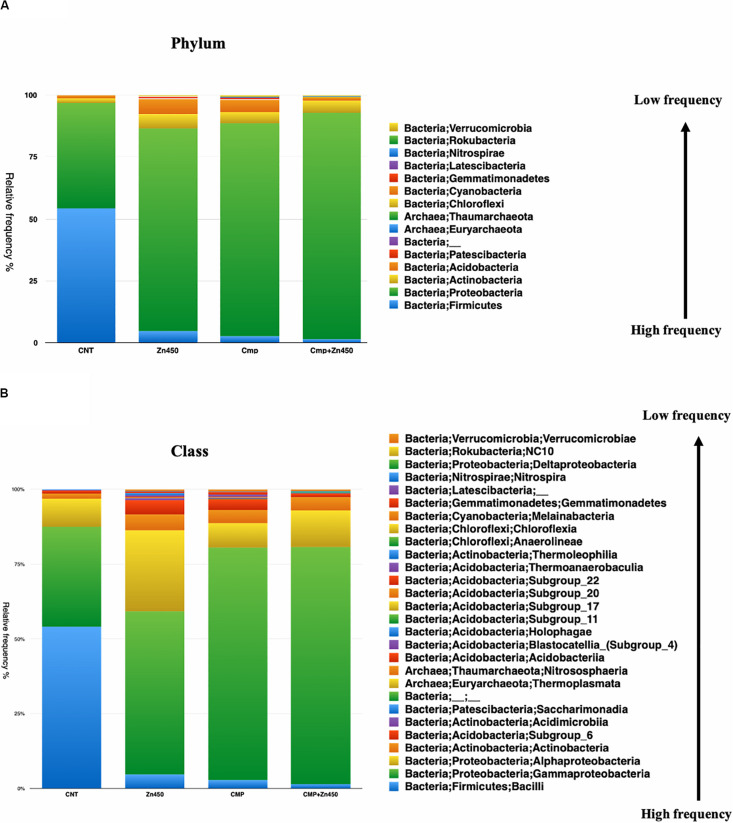
Overview of the relative frequency of root microbiome with respect to **(A)** phylum or **(B)** class under different experimental conditions.

The taxa bar plot was carried out at three different hierarchic levels: phylum, class and family. At phylum level (Figure 3A), the results revealed the presence of 15 phyla. The most abundant were Proteobacteria and Firmicutes.

The Firmicutes phylum was predominant in CNT and its relative abundance was reduced in the other three experimental groups, whilst, Proteobacteria phylum increased its relative abundance as well as Actinobacteria and Acidobacteria phyla. All the other phyla showed a relative low frequency and were strongly influenced by Zn addition in the case of the Zn450 (e.g., Patescibacteria) or CMP + Zn450 (e.g., Chloroflexi). At the class level, Gammaproteobacteria (Figure 3B) dominated the CMP rhizosphere microbiome, and their relative abundance was not affected by Zn addition. On the contrary, the CNT thesis was characterized by a high relative frequency of Bacilli, which, in turn, was negatively affected by Zn addition (Zn450). In this case, the Alphaproteobacteria, Blastocatella, and Holophagae (belonging to the phylum of Acidobacteria) classes increased greatly compared to the CNT. When the CMP and CMP + Zn450 groups were considered, several phyla and classes (absent in the other groups) were present, such as Anaerolineae, Thermoleophilia and other classes belonging to the Acidobacteria phylum (Subgroups 6, 17, and 22).

**FIGURE 4:**
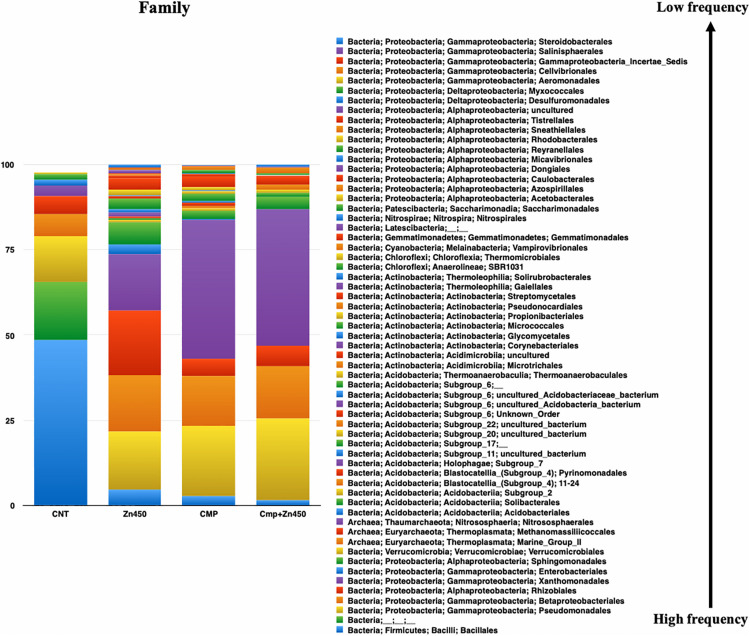
Overview of the relative frequency of root microbiome with respect to family, under different experimental conditions.

At the family level (Figure 4), 62 OTUs were identified, and the most abundant were represented by Xanthomonadales, Pseudomonadales, and Betaproteobacteriales (belonging to the Phylum Proteobacteria, class Gammaproteobacteria), Bacillales (belonging to the Phylum Firmicutes, class Bacilli), and Rhizobiales (belonging to the Phylum Proteobacteria, class Alphaproteobacteria). The rhizosphere microbiome of CNT was characterized by the highest relative frequency of Bacillales (phylum Firmicutes, class Bacilli), and the lowest of Xanthomonadales, while other families were represented at low relative frequency. Moreover, some families were particularly enriched in some specific rhizosphere microbiome; even though CMP amendment and/or Zn addition deeply modified the rhizosphere community; the bacteria families, initially characterizing the CNT rhizosphere microbiome were still present in the other experimental group, but with different relative frequency (Rhizobiales, Verrucomicrobiales, Pseudomonadales, Betaproteobacteriales, Xanthomonadales, and Bacillales). In particular, Zn alone favored the presence of Micrococcales, Pyrinomonadales, and Dongiales classes.

In the case of CMP group, the most represented families were Xanthomonadales, Pseudomonadales and Betaproteobacteriales, and they were not affected by the Zn addition (CMP + Zn450 vs. CMP). However, CMP amendment favored the presence of Aeromonadales, Desulfuromonadales, Tistrellales, whilst Zn addition and CMP amendment (CMP + Zn450) modified the rhizosphere microbiome favoring the presence of Corynebacteriales, Gaiellales, Salinisphaerales, and Pseudonocardiales families.

##### Qualitative and quantitative analysis of rhizobacteria communities

A qualitative and quantitative analysis of the rhizosphere microbiome was carried out in order to evaluate the differences, in terms of the classes or families, that were attributable to CMP amendment and/or Zn addition. In particular, the Venn analysis (Table 5) revealed that the experimental group with the highest number of unique classes was that of CMP (7 bacteria classes), followed by the other three groups in decreasing order: CMP + Zn450 > Zn450 > CNT (4, 1 and 0 unique classes, respectively). A large part of the bacteria classes was shared among the different groups (6; Table 5).

**TABLE 5:** Venn table of the shared or unique OTUs among experimental groups.

**Experimental theses**	**Number of shared OTUs**	**OTUs**
CNT; Zn450; CMP;	6	Alphaproteobacteria
CMP + Zn450		Verrucomicrobiae
		Acidobacteria; Subgroup_6
		Actinobacteria
		Bacilli
		Gammaproteobacteria
CNT; Zn450; CMP + Zn450	1	Acidimicrobiia
Zn450; CMP;	2	Nitrospira
CMP + Zn450		Gemmatimonadetes
CNT; Zn450	1	Saccharimonadia
Zn450; CMP	4	Acidobacteriia
		Thermoplasmata
		Blastocatellia
		Thermoanaerobaculia
CMP; CMP + Zn450	1	Anaerolineae
Zn450	1	Holophagae
CMP	7	Acidobacteria; Subgroup_19
		Acidobacteria; Subgroup_18
		Deltaproteobacteria
		Acidobacteria; Subgroup_22
		Acidobacteria; Subgroup_24
		Acidobacteria; Subgroup_23
		Acidobacteria; Subgroup_17
CMP + Zn450	4	Acidobacteria; Subgroup_13
		Acidobacteria; Subgroup_12
		Thermoleophilia
		Acidobacteria; Subgroup_11

**FIGURE 5:**
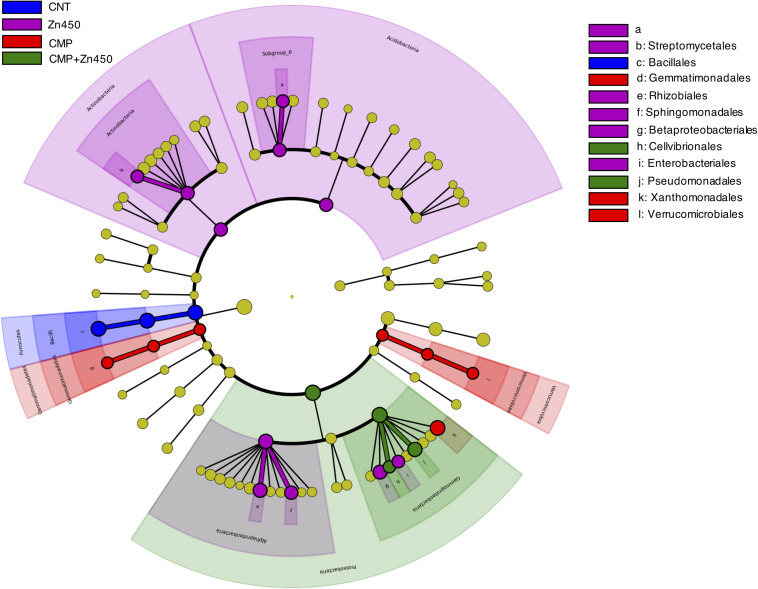
Biomarkers of the rhizosphere microbiome of different experimental groups. LEfSe analysis was used to validate the statistical significance and the size effect of the differential abundances of the taxa of bacterial rhizosphere community of N12 poplar saplings (Kruskal–Wallis and Wilcoxon rank-sum *p* < 0.05 and LDA score > 3.5). In the cladogram, the taxonomic classification shows class, order, and family of the different experimental groups, while the genus is represented, with different letters, on the right side of the figure.

In order to investigate whether some bacteria phylum, class or family was a potential marker of the diverse rhizosphere microbiome, a LEfSe analysis was performed (Figure 5). Seventy-five root Absolute Sequence Variants (ASVs) were identified with LEfSe (*p* < 0.05, log10 LDA score > 3.5) among the different experimental groups. In particular, members belonging to Bacillales family (belonging to Phylum Firmicutes, class Bacilli) may be considered a marker of CNT microbiome, whilst Actinobacteria (Streptomycetales) and Acidobacteria (Subgroup_6) were for Zn450. The CMP and CMP + Zn450 rhizosphere microbiome were characterized by the presence of Gemmatimonadales and Verrucomicrobiales, or Pseudomonadales and Cellvibrionales (Gammaproteobacteria) classes, respectively.

#### Biodiversity of Fungal Rhizosphere Microbiome

ITS sequencing generated 1,184,489 gene sequences and, after quality filtering, denoising and removal of chimeric sequences, 1,015,382 genes sequences remained. The ASV was 718 and the rarefaction curves are reported in Supplementary Figure S2. The α biodiversity was investigated for rhizosphere fungal communities and, in general, both CMP amendment and/or Zn addition altered them. In particular, the lowest α diversity was observed in the case of CMP + Zn450 (Table 6).

**TABLE 6:** Kruskal-Wallis pairwise comparison of two α diversity indices **(A)** 1256 Shannon, **(B)** Simpson, and **(C)** Observed Otus among the experimental groups.

**(A) Shannon index**		
**Thesis 1**	**Thesis 2**	***p*-value**

CNT (*n* = 3)	CMP (*n* = 3)	0.512
**CNT (*n* = 3)**	**CMP + Zn450 (*n* = 3)**	**0.049**
**CNT (*n* = 3)**	**Zn450 (*n* = 3)**	**0.049**
**CMP (*n* = 3)**	**CMP + Zn450 (*n* = 3)**	**0.049**
CMP (*n* = 3)	Zn450 (*n* = 3)	0.512
**CMP + Zn450 (*n* = 3)**	**Zn450 (*n* = 3)**	**0.049**

**(B) Simpson index**		
**Thesis 1**	**Thesis 2**	***p*-value**

CNT (*n* = 3)	CMP (*n* = 3)	0.275
**CNT (*n* = 3)**	**CMP + Zn450 (*n* = 3)**	**0.049**
**CNT (*n* = 3)**	**Zn450 (*n* = 3)**	**0.049**
**CMP (*n* = 3)**	**CMP + Zn450 (*n* = 3)**	**0.049**
CMP (*n* = 3)	Zn450 (*n* = 3)	0.512
**CMP + Zn450 (*n* = 3)**	**Zn450 (*n* = 3)**	**0.049**

**(C) Observed OTUs index**		
**Thesis 1**	**Thesis 2**	***p*-value**

CNT (*n* = 3)	CMP (*n* = 3)	0.275
**CNT (*n* = 3)**	**CMP + Zn450 (*n* = 3)**	**0.049**
**CNT (*n* = 3)**	**Zn450 (*n* = 3)**	**0.049**
**CMP (*n* = 3)**	**CMP + Zn450 (*n* = 3)**	**0.049**
CMP (*n* = 3)	Zn450 (*n* = 3)	0.512
**CMP + Zn450 (*n* = 3)**	**Zn450 (*n* = 3)**	**0.049**

*In bold are indicated the statistically significant differences (*p* < 0.05).*

Furthermore, the comparison of Observed OTUs and Simpson indices revealed statistically significative differences among all treatments (*p* < 0.05), with the exception of the comparison between CMP and Zn450. In addition, each treatment significantly affected the Shannon indices (*p* < 0.05) when compared with CNT, with the exception of CMP. Although for this index, the comparison between CMP and Zn450 was not statistically significant (Table 6). Correlation analyses revealed that the Shannon index and N 12 sapling biomass were not statistically correlated (*r* = 0.22; *p* < 0.05). On the contrary, the Pearson coefficient highlighted that Shannon diversity and Zn addition were strongly negatively correlated (*r* = −0.82; *p* < 0.005). A similar result in terms of correlation was obtained estimating the correlation between Zn addition and Chao 1 (*r* = −0.77; *p* < 0.005), or Simpson index (*r* = −0.77; *p* < 0.005).

The b diversity was calculated in order to highlight the differences among the experimental groups using Dice metrics. The results (Table 7) highlighted that all the groups were well distinguished and the distance among them was statistically significant (*p* ≤ 0.1).

**TABLE 7:** The pairwise PERMANOVA tests whether Dice distances between samples within the same experimental group are more similar to each other than they are to samples from the other groups.

**Thesis 1**	**Thesis 2**	**Sample size**	**Permutations**	**pseudo-F**	***p*-value**	***q*-value**
**CNT**	**CMP**	**6**	**999**	**19.09**	**0.101**	**0.109**
**CNT**	**CMP + Zn450**	**6**	**999**	**27.55**	**0.102**	**0.109**
**CNT**	**Zn450**	**6**	**999**	**19.17**	**0.090**	**0.109**
**CMP**	**CMP + Zn450**	**6**	**999**	**42.28**	**0.097**	**0.109**
**CMP**	**Zn450**	**6**	**999**	**42.29**	**0.109**	**0.109**
**CMP + Zn450**	**Zn450**	**6**	**999**	**108.03**	**0.101**	**0.109**

*Statistically significant values are indicated in bold (*p* ≤ 0.1).*

##### Fungal community composition

**FIGURE 6:**
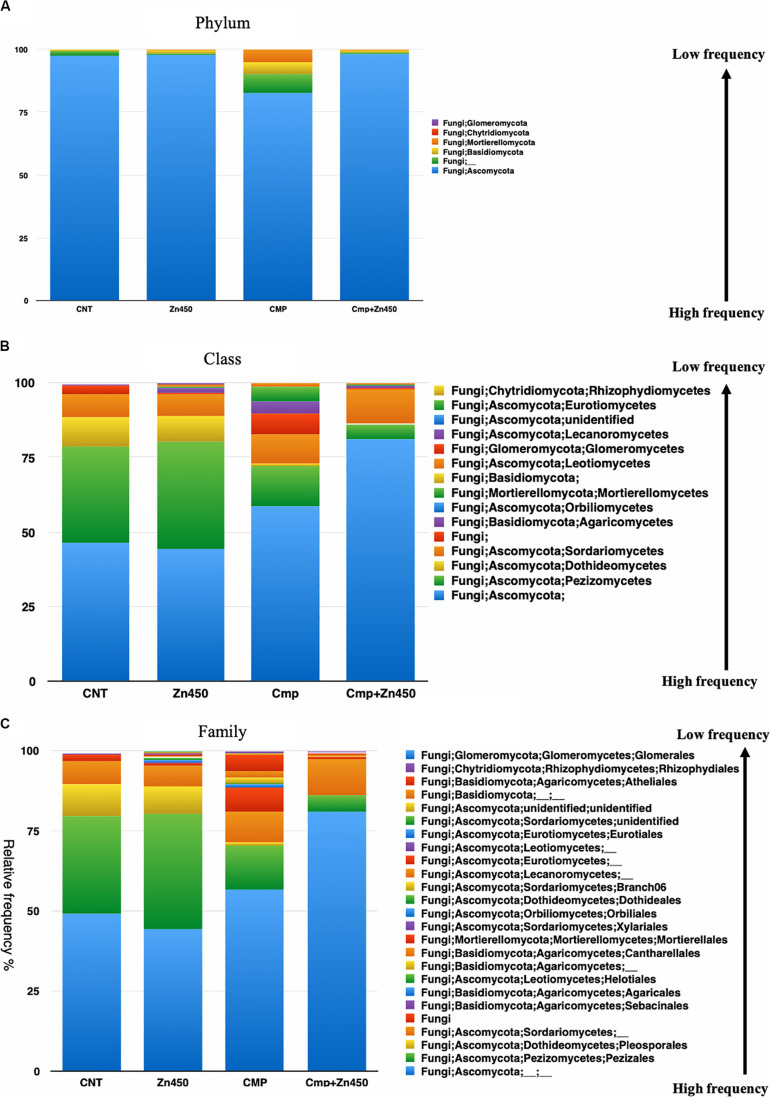
Overview of the relative frequency of root surface fungi with respect to **(A)** phylum, **(B)** class or **(C)** family under different experimental groups.

The taxa bar plot was reported at three hierarchic levels: phylum, class and family. At phylum level (Figure 6A) Ascomycota, Basidiomycota, Mortierellomycota, Glomeromycota, and Chytridiomycota phyla characterized the rhizosphere fungal community. Ascomycota phylum was the dominant one, however, the CMP experimental group showed also a relative high frequencies of Basidiomycota and Mortierellomycota phyla respect to the other experimental groups.

At the class level (Figure 6B), the differences in relative frequency between CMP amended or unamended soil were highly evident. Zinc addition greater affected the fungal microbiome rhizosphere classes of CMP amended soils compared to those of unamended soil. In fact, only the relative frequency of the less abundant classes: Lecanoromycetes, Agaricomycetes and Leotiomycetes, belonging to the phylum of Ascomycota, were affected in the case of Zn450 and increased with respect to the CNT. In CMP + Zn450, the Zn addition deeply modified the fungal rhizosphere community, mainly for the most abundant classes (e.g., Pezizomycetes, Sordariomycetes, and Dothideomycetes; CMP vs. CMP + Zn450), but also for the less represented ones (e.g., Agaricomycetes, Pezizomycetes, and Mortierellomycetes).

This trend was confirmed when the third hierarchic level was considered (Figure 6C). The most represented families in CNT were not affected by the addition of Zn (CNT vs. Zn450; e.g., Pezizales, Pleosporales, etc.) the less represented families slightly increased, as in the case of those belonging to Lecanoromycetes and Leotiomycetes (Helotiales). CMP amendment slightly augmented the frequency of some families less represented in the CNT group (CNT vs. CMP). On the contrary, when the fungal rhizosphere of CMP and CMP + Zn450 were considered a sensible modification of the class composition, due to Zn addition, was observed. In particular, the relative frequency of Mortierellales (Tremellomycetes) and Cantharellales (Agaricomycetes) was negatively affected.

##### Qualitative quantitative analysis of fungi rhizosphere community

The fungi community was investigated from qualitative and quantitative points of view, aiming to identify those fungal families that were specifically associated to the treatments of the different experimental groups. At the family level, the Venn analysis (Table 8) revealed that three, two, two, and five fungal families were specific to CNT, Zn450, CMP, and CMP + Zn450, respectively.

**TABLE 8:** Venn table of the shared or unique OTUs among groups.

**Experimental groups**	**Number of shared OTUs**	**OTUs**
CNT; ZN450; CMP;	5	Cantharellales
CMP + ZN450		Mortierellales
		Agaricales
		Pezizales
		Pleosporales
CNT; ZN450; CMP	5	Glomerales
		Leotiomycetes;__
		Sebacinales
		Helotiales
		Agaricomycetes;__
CNT; ZN450; CMP + ZN450	1	Xylariales
CNT; ZN450	1	Sordariomycetes;__
CNT; CMP	2	Dothideales
		Orbiliales
ZN450; CMP	1	Lecanoromycetes;_
CMP CMP + ZN450	1	Sordariomycetes;_
CNT	3	Sordariomycetes; Branch07
		Lecanoromycetes;__
		Eurotiomycetes
ZN450	2	Sordariomycetes; Branch11
		Sordariomycetes; unidentified
CMP	2	Sordariomycetes; Branch15
		Rhizophydiales
CMP + ZN450	5	Agaricomycetes;_
		Eurotiales
		Atheliales
		Leotiomycetes;_
		Sordariomycetes; Branch19

In particular, the CMP group was enriched by fungal families belonging to the Sordariomycetes and Rhizophydiales classes; whilst Agaricomycetes, Eurotiales, Atheliales, and Leotiomycetes were present when the CMP + Zn450 was considered. Interestingly, five families, Cantharellales, Mortierellales, Agaricales, Pezizales and Pleosporales, were present in all experimental theses.

**FIGURE 7:**
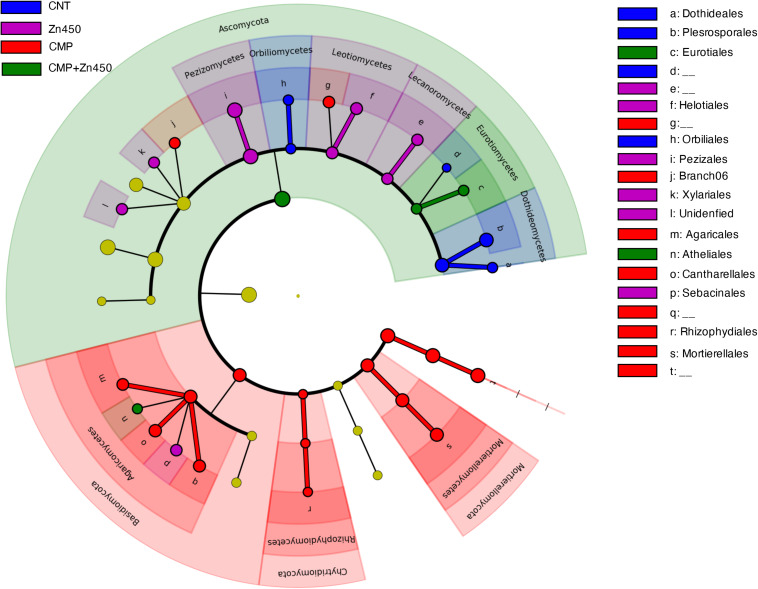
Biomarkers of the rhizosphere microbiome of the different experimental groups. LEfSe analysis was used to validate the statistical significance and the size effect of the differential abundances of the taxa of fungal rhizosphere community of N12 poplar saplings (Kruskal–Wallis and Wilcoxon rank-sum *p* < 0.05 and LDA score > 3.5). In the cladogram, the taxonomic classification shows class, order, and family of the different experimental groups, while the genus is represented, with different letters, on the right side of the figure.

A LEfSe analysis (Figure 7), as in the case of bacterial communities, was performed to identify hypothetic taxonomic markers (phyla, classes or families) of the specific analyzed experimental groups.

Only 47 root ASVs (LEfSe. *p* < 0.05, log10 LDA score > 3.5) were enriched among the different experimental groups. Dothideales and Pleosporales families (both belonging to the phylum of Ascomycota, class Dothideomycetes) can be considered as markers of the CNT; Rhizophydiales (phylum Chytridiomycota. class Rhizophydiomycetes), Mortierellales (phylum Mortierellomycota, class Mortierellomycetes), Cantharellales and Agaricales (both belonging to the phylum of Basidiomycota class Agaricomycetes) were markers of CMP, while Eurotiales (phylum Ascomycota, class Eurotiomycetes) was maker of CMP + Zn450. No one specific marker was identified for Zn450 group.

## Discussion

The N12 black poplar clone was previously selected as being highly tolerant of two heavy metals: Cu and Zn (Castiglione et al., 2009). Moreover, research conducted by us during the last 15 years has revealed its remarkable capacity for growth and phytostabilization in differentially contaminated soils (Cu, Fe, and Pb), and also for phytoextraction of Cd and Zn (Baldantoni et al., 2014).

The present study was conducted to explore the dynamics of the rhizosphere microbiome associated to N12 saplings in the context of HM phytoremediation. As expected, N12 poplar saplings grew normally under all experimental conditions. In fact, they had a normal biomass and showed no signs of toxicity in the presence of Zn. Moreover, they accumulated high amounts of the metal, confirming, once more, its particular suitability to cultivation on HM polluted soils. Based on the percentage and type of CMP added to the pot soil, the amendment exerted positive, albeit limited effects on the growth and biomass, mainly affecting the leaves, as well as on Zn removal from the soil, whilst they had no measurable effect on Zn soil bioavailability and its uptake into the plants.

Yields of different trees or crops, as well as plant biometric and morphometric parameters, can be improved as a result of soil amendments, as in the case of maize and barley (Hernández et al., 1991; Roca-Pérez et al., 2009; Lamari and Strelkov, 2010), sunflower (Moreno et al., 1997), wheat (Lamari and Strelkov, 2010), fast growing trees (Madejón et al., 2016). It is known that CMPs of different origins, containing considerable amounts of nutrients, *humus* and microorganisms (Farrell et al., 2010), have beneficial effects on metabolism of soil biota, on dynamic of nutrient uptake and on physical soil properties, as well as on the enzymatic activity involved in phosphorus and nitrogen cycles, that enhance plant growth and productivity (Semida et al., 2014). Some studies (Sotta et al., 2019) suggest that Zn alone interferes with leaf morphology, and, in general, retardation in growth rate or biomass production, as well as the effects on plant morphology, might be due to interference of HMs with the processes of plant mineral uptake (Kidd et al., 2017; Kushwaha et al., 2018).

In our study, CMP alone, or in combination with Zn, had a positive and significant effect on the morphological parameters analyzed, especially on those of leaves. In fact, the increase of biomass and leaf expansion higher than CNT, is a clear indication that CMP may be protective with regard to Zn uptake and toxicity.

Both the CNT and CMP soils used in the experiment did not show evident differences in pH and nutrient content (e.g., Zn total or bioavailable fractions). The CMP supplied can significantly modify physical and chemical properties of the soil of CMP group, and in fact it slightly increased organic matter content and CEC respect to CNT. However, the amendment had no effect on Zn bioavailability and uptake by the poplar saplings. On the contrary, it favored Zn soil stabilization. The hazard of inorganic contaminants, such as metals and metalloids, arises from their absorption and accumulation in the cells. In fact, although Zn is an essential micronutrient for all living organisms, an excess of it can be toxic (Sharma et al., 2013).

Zn mobility in soil may be altered by the presence of some oxidized forms of Fe, Ca, Al, Mn, and P, and also by organic matter. Soil organic amendments can reduce solubility and mobility of metals, immobilizing them and/or decreasing their leaching (Huang and Salt, 2016). Various sorption processes, adsorption to mineral surfaces, formation of stable complexes with organic ligands, surface precipitation and ion exchange, co-precipitations can contribute to reduced mobility of the contaminants (Colugnati et al., 1995). Moreover, these sorption/dissolution processes are influenced by pH, CEC, redox potential, soil constituents, and in general, a single mechanism does not explain the immobilization of elements into the soil matrix (Kabata-Pendias, 2000).

However, the supply organic amendments to the soil can modify CEC, which, in turn, improves soil trace and oligo element (e.g., Zn) retention. Some studies have revealed that CMP, applied alone or in combination with others (e.g., biochar), increased the CEC of the soil due to the input of stabilized organic matter, which is rich in functional groups (such as carboxylic and phenolic acid groups) released into the soil exchange sites (Ouédraogo et al., 2001; Yang et al., 2019). Paradelo et al. (2011) analyzed in detail metallic chelates of the soil, observing that organic matter can play a key role in the immobilization of trace elements by forming stable compounds with them. Other studies have revealed that pH increased after the CMP soil addition, with a consequent reduction of trace element bioavailability (Pérez-De-Mora et al., 2006; Baker et al., 2011; Zhou et al., 2011; Strachel et al., 2017). However, the significance of the effects on metal bioavailability might be due to the type and amount of CMP added. Taiwo et al. (2016) observed that the higher the CMP dose added to the soil, lower was the Zn bioavailability.

In our study, we found that N12 saplings were able to accumulate Zn in all of the organs analyzed (roots, stems and leaves): the highest concentration of Zn was observed in the roots followed by the stems and leaves. The binding of contaminants at root cell walls, or their accumulation and storage into the vacuole, are recognized avoidance mechanisms for trace metals in plants (Krzesłowska, 2011). Moreover, in our study, Zn uptake decreased with the addition of CMP, mainly in leaves, which, in turn, increased their size and biomass. In general, we found that the addition of both Zn and CMP to the soil did not affect Zn accumulation, probably because CMP is able to decrease Zn availability throughout bio-accumulation and bio-adsorption processes.

Our results are consistent with previous findings where the application of CMP did not increase Zn concentrations in the plant organs or it decreased it slightly (Zhou et al., 2017). Soares et al. (2019) reported that broad bean plants accumulated increasing levels of Zn in the treatments with low or moderate CMP addition to the soils; in contrast, its content decreased significantly in the different organs of the plants with higher soil CMP additions. The authors postulated that this phenomenon might be caused by the lower available Zn content into the soil due to CMP sequestration.

Although it is known that the *P. nigra* N12 clone is suitable for Zn phytoextraction (Castiglione et al., 2009; Baldantoni et al., 2014), there is no information on the rhizosphere microbiome responses to Zn addition and/or CMP amendment of soil, or the role of microbiome in the phytoremediation processes of this black poplar clone. In order to shed light on these processes and to assess variations caused by Zn soil addition and/or CMP amendment, we analyzed the rhizosphere microbiome of N12 saplings because it may contribute to plant growth, health (Richter-Heitmann et al., 2016) and metal tolerance. Our interest comes from several studies which have clearly established that the rhizosphere corresponds to the plant-soil compartment harboring the highest microbe richness diversity (Guo et al., 2019; Latini et al., 2019; Brereton et al., 2020). Soil rhizospheres are very complex matrices in relation to their bacterial and fungal communities, which can be readily analyzed in terms of diversity and composition, by deep sequencing (Burges et al., 2017; Fan et al., 2018). Furthermore, species richness diversity and their abundance levels may be inferred by the α-diversity indexes. Our experiment showed that the microbiome assembly during phytoremediation was positively influenced not only by Zn contamination, but also by CMP soil amendment. Furthermore, the CMP rhizosphere microbiome showed the highest α-diversity, followed by CMP + Zn450, Zn and CNT. We also detected a slight alteration of microbial diversity by Zn addition and an increase attributable to CMP amendment. Firmicutes, Proteobacteria, Acidobacteria and Actinobacteria were the dominant phyla of the rhizosphere communities of all N12 poplar saplings, although some differences were observed among the different groups.

Firmicutes and Proteobacteria and, to lesser extent, Actinobacteria and Acidobacteria dominated the CNT rhizobacterial microbiome. Firmicutes phylum was predominant in CNT, and the species, belonging to it, have been widely studied and recognized as “plant-beneficial” bacteria (Mendes et al., 2013). LEfSe analysis, which was mainly used to identify significantly enriched bacterial taxa among all analyzed microbiome rhizospheres, confirmed, once more, that the Bacillales family is potential biomarker of the CNT. Whereas the presence of Proteobacteria, which has been considered to be an indicator of nutrient-rich soils, indicated that a good quality and rich soil was used here.

Information about the effects of Zn pollution on the rhizobacteria communities of the poplar is very few. In general, HMs have a toxic impact on microbe communities when their content is high in the soil. In fact, it has been reported that a high soil metal contamination reduces the species number of microbes, and also bacterial and fungal diversity, and this it was particularly true for bulk soils (Rajapaksha et al., 2004; Stefanowicz et al., 2008; Azarbad et al., 2013).

Root secretions can significantly modulate bioavailability of metals in the soil, including their concentration and toxicity, and, consequently, affect the microbial communities of the rhizosphere (Chen et al., 2003; Zhou et al., 2011). In our study, a detrimental effect of Zn addition was not observed at the whole bacterial community level, however its addition to the soil resulted in a differential abundance of particular taxa. Compared to the CNT rhizosphere microbiome, Proteobacteria, Acidobacteria and Actinobacteria phyla increased and that of Patescibacteria became apparent. Several genomic and metagenomic studies have shown that members of the Patescibacteria “superphylum” showed reduced metabolic capabilities that likely limit their cultivation (Kantor et al., 2013; Rinke et al., 2013). This superphylum is also involved in hydrogen production, sulfur cycling (Wrighton et al., 2012; Kantor et al., 2013), and anaerobic methane oxidation (Peura et al., 2012), and it is responsible for the removal of conventional contaminants from the soil, including HMs and/or antibiotics (Wang et al., 2018; Yang et al., 2019).

Substantial changes were observed in the rhizobacterial community structure of N12 poplar saplings when grown on soils amended with CMP. Our results were consistent with other studies that have reported the effect of CMP on soil biodiversity (Hartmann et al., 2015; Ye and Tang, 2016), and its alteration during the phytoextraction process. Organic amendments can modify soil physico-chemical properties and provide nutrients and vast amounts of microorganisms, the composition of which largely depends on the source material (Sun et al., 2015; Yang et al., 2019). Thus they can create conditions that are favorable for the growth of microorganisms that are beneficial to plants, while simultaneously inhibiting others (Husson, 2013; Azarbad et al., 2015; Ye and Tang, 2016). The Proteobacteria, Acidobacteria and Actinobacteria phyla displayed greater abundance in the N12 rhizosphere microbiome of CMP amended soils compared to CNT. The abundance of these phyla might be explained by the increase in soil carbon content due to the addition of organic matter. According to the trophic life histories of soil bacteria, these phyla include “copiotrophic” bacteria (r-strategists), which use labile carbon for their metabolism and growth, and this allows them to grow faster in nutrient-rich environments (Fierer et al., 2007; Trivedi et al., 2013; Deng et al., 2020).

Although the LEfSe analysis indicated that Gemmatimonadales and Verrucomicrobiales, or Pseudomonadales and Cellvibrionales are biomarkers of CMP and CMP + Zn450, respectively, all rhizobacterial communities of N12 poplar saplings grown on CMP amended soils were quite similar when Zn was added to the soil. Therefore, this suggests that Zn addition changes only weakly the structure of the microbial community when CMP is added to the soil. At phylum level, the CMP + Zn450 rhizosphere was characterized by the presence of Chloroflexi, previously identified as green non-sulfur bacteria, that includes a relatively understudied bacterial phylum with diversified metabolism and, in some cases, with a strong resistance to HMs (Gremion et al., 2003; Azarbad et al., 2015). Bacterial taxa belonging to this phylum were reported to be prevalent in nutrient poor soils (Will et al., 2010), in oligotrophic ecosystems, such as soils of high-elevation regions where vegetation is patchy, or decreases in the presence of high levels of nitrogen (Ding et al., 2013). In our study, the relative abundance of Chloroflexi increased in Zn450 + CMP soil, confirming, to some extent, the results reported for the rhizosphere of *Elsholtzia splendens* Nakai, a Cu-tolerant plant native to China, where a relationship between the abundance of Chloroflexi and a higher level of carbon source was detected in the rhizosphere soils, supporting its sensitivity to soil nutrient content (Jiang et al., 2016).

Correlation analysis was performed between microbial biodiversity indices and Zn content in the different organs of N12 poplar saplings, revealing that biodiversity was significantly correlated with the metal content in roots and stems, the two main accumulating organs, and even with the leaf area. This suggests that greater bacterial diversity stimulates Zn absorption by changing the physico-chemical characteristics of the rhizosphere, and, at the same time, impacting nutrient uptake and, consequently, plant growth.

Most of our current knowledge on rhizomicrobial diversity relates to bacterial communities, while information on fungal communities is quite scarce (Cicatelli et al., 2019). Studying and understanding fungal communities is of paramount importance since fungi comprise a major portion of the biomass and biodiversity of the Earth soil. In fact, fungi play crucial roles in maintaining soil processes which affect the functioning of the largest part of the ecosystems (Narendrula-Kotha and Nkongolo, 2017). For this reason, we also investigated the effects of Zn addition on the fungal rhizosphere communities of the N12 poplar saplings grown on CMP amended soil. Although the α-diversity of the rhizobacterial community increased in all experimental settings, compared to the CNT, in the case of the fungal community the trend was the opposite, with diversity being significantly reduced in CMP, Zn450 and CMP + Zn450 soils. The α-diversity of fungal rhizosphere was negatively correlated with the Zn content in the soils of N12 poplar saplings; in fact, this occurred in both Zn450 and Zn450 + CMP soils. This suggest that Zn soil addition selected only metal tolerant fungal communities (lower biodiversity), which, in turn, would improve the capacity of the plant to take up and accumulate Zn and counteract its negative effects. Contrary to what we observed, Kamal et al. (2010) found that species richness and diversity, represented by the Shannon–Wiener index, increased at moderate levels of soil pollution, while it was reduced at the higher ones, simulating in this way a homeostatic response. The increment of fungal biodiversity at HM moderate concentrations could also be a stress response, whereby fungal ecotypes better adapted to unpolluted soil, allow other fungi (probably less competitive in unstressed soils but better adapted to heavy metals) to colonize the roots and complete their life cycles. Few studies have reported the negative effects of metals on fungal growth and reproduction (Azevedo and Cássio, 2010; Goupil et al., 2015). These studies showed that metal toxicity varies on the basis of the fungal species, type of metal and its concentration, nutrient availability, and plant species diversity.

In our study the presence of Zn did not affect the more represented classes and families, however, Zn addition reduced negatively affected the number of the less represented OTUs represented in CNT and CMP. Several studies revealed that the fungal rhizosphere microbiome can sustain plants during their life cycles, especially in stressful conditions, through mechanisms such as chelation with organic ligands, transportation out of the cells, and biotransformation of the ions to less bioavailable or less toxic metal species. Vascular plants host a great variety of fungi in all of their organs. In addition, being susceptible to soil-borne pathogens, plant roots are also colonized by non-pathogenic or mutualistic fungi such as endomycorrhizal fungi (AMF), ectomycorrhizal fungi (EMF), and dark septate endophytes (DSE). The AM fungi comprise about 150 species of zygomycetous fungi, and EM fungi include about 6.000 species that are primarily Basidiomycetes, along with a few Ascomycetes and Zygomycetes (Saikkonen et al., 1998).

Differently to that observed in the case of rhizobacteria community, the CNT fungal rhizosphere community was not modified by soil Zn addition (Zn450). For all of the other experimental groups, Ascomycota and Basidiomycota phyla were abundantly represented (about 95%). Only the less represented classes of CNT, such as Lecanoromycetes, Agaricomycetes and Leotiomycetes, belonging to the phylum of Ascomycota, increased in the case of Zn450. Fungi, especially those belonging to Ascomycetes and Basidiomycetes, are able to degrade very complex organic compounds including cellulose and lignin, but many of them also live as root symbionts (mycorrhizas) and obtain simple sugars from their plant partners (van der Heijden et al., 2015). The range of Zn toxic concentrations among fungi is highly variable (generally from 10 up to 500 mg L^–1^) depending on the species, strains, or even the type of growth media (de Oliveira and Tibbett, 2018). Høiland (1995) tested metal toxicity in Basidiomycota, and found Cd to be very toxic, whilst Zn affected only moderately this genus. The large part of the fungal taxa identified in our study was unaffected by Zn soil addition. A greater effect on the fungal rhizosphere microbiome was due to the CMP amendment. Wu et al. (2019), found that the same three fungal phyla (Basidiomycota, Ascomycota, and Mortierellomycota), which predominated in CNT, Zn450, CMP, and CMP + Zn450 groups, were not significantly affected by CMP, except for a slight increase in abundance of Ascomycota.

The fungal community composition of CNT and CMP differed in relation to four classes: Pezizomycetes, Dothideomycetes, Agaricomycetes, and Mortierellomycetes. The CMP reduced the relative frequencies of the first two classes (both belonging to Ascomycota phylum), whilst it increased in the case of Agaricomycetes (Basidiomycota phylum) and Mortierellomycetes (Mortierellomycota phylum). Furthermore, these four classes were also the ones that were more sensitive to Zn addition in the case of CMP + Zn450. In fact, when CMP + Zn450 was compared to CMP, the relative frequency of Agaricomycetes and Mortierellomycetes, which are characteristic of the CMP, was reduced by the Zn addition. These differences in the relative abundance of specific OTUs, due to the different ecologic driven force (CMP and/or Zn), were also investigate trough LEfSe analyses in order to identify specific markers in each experimental group. The CNT fungal rhizosphere microbiome was characterized by the presence of Dothideales and Pleosporales families (both belonging to the phylum of Ascomycota, class Dothideomycetes), whilst CMP was characterized by the presence of Rhizophydiales (phylum Chytridiomycota, class Rhizophydiomycetes), Mortierellales (phylum Mortierellomycota, class Mortierellomycetes), Cantharellales and Agaricales (both belonging to the phylum of Basidiomycota class Agaricomycetes). In particular, Eurotiales family (phylum Ascomycot, class Eurotiomycetes) was identified as maker for CMP + Zn450. This family was recognized as a taxon able to tolerate organic pollution; in fact, Harcourt and State (2014) observed that Eurotiales family (Ascomycota phylum) was able to thrive in extreme environments, such as those polluted by crude oil. It is noteworthy, that in a few studies of the ability of fungi to grow in polluted environments, Ascomycetes are highly represented compared to Basidiomycetes (Kozdrój and Van Elsas, 2000; Obire and Anyanwu, 2009; Thion et al., 2012). Hartikainen et al. (2012) also recognized that Basidiomycetes tolerated better lower Zn concentrations than Ascomycetes and Zygomycetes, whilst the opposite was true at higher Zn concentrations. This finding indicates that changes in fungal communities occur when different amounts of Zn are added to the soil and they are also directly related to its increasing concentrations of the metal (Hartikainen et al., 2012). In our study, as expected, soil CMP addition modified the fungal communities and increased the biomass of the saplings especially of leaves, however, this improvement was not positively correlated with fungal diversity of the fungal rhizosphere microbiome, but, probably, related to the input of organic matter. In contrast, Zn addition negatively affected the fungal rhizosphere microbiome in terms of biodiversity; this was expected given the selective pressure exerted by high Zn doses added to the soils in our experimental groups.

## Conclusion

Phytoremediation is one of the most widely studied green technologies for soil and water reclamation. In the last 20 years, many studies have focused on the identification of the best plant species to accumulate, extract, degrade or volatilize, inorganic or organic contaminants in specific pedoclimatic conditions. However, although these studies have improved knowledge and yielded interesting results, a thorough understanding of the interactions between plants and soil/rhizosphere microorganisms, and of roles that microbes play in phytoremediation processes, is still far away. In addition, soil amendment with CMP represents both a source of organic matter and of microorganisms, both of which are useful to improve the physico-chemical soil properties (often impoverished by metal pollution) and also increase the biodiversity. The resistance and resilience of soil microbiomes in turn can improve plant tolerance to contaminants and promote their growth. In this study, CMP amendment was able to improve biomass of N12 poplar saplings, in particular that of leaves, and, mostly, it counterbalanced the negative effects of Zn soil addition on their biomass. A greater biomass due to CMP amendment allowed a lower Zn accumulation in the different plant organs preserving plant health and, at the same time, improving the total Zn content in the N12 poplar saplings. Our experimental results demonstrated that CMP amendment induced different effects on bacterial and fungal rhizosphere microbiome; in fact, in the case of bacteria, CMP amendment deeply affected the rhizosphere microbiome, which in turn was more resilient to the presence of Zn. In contrast, in the case of fungal rhizosphere microbiome, CMP amendment strongly modified the community structure. Moreover, the Zn addition mainly affected the fungal rhizosphere microbiome of saplings grown on CMP amended soil. Our study confirmed that the rhizosphere microbiome is characterized by a complex network of relationships through which plants obtain several advantages. In our opinion, further studies on phytoremediation should be focused on these relationships with the goal of understand how these networks may improve phytoremediation effectiveness and opening the route to phytoremediation phase 2.0.

## Data Availability Statement

The datasets presented in this study can be found in online repositories. The names of the repository/repositories and accession number(s) can be found at: https://www.ebi.ac.uk/metagenomics/, PRJEB39028 and https://www.ebi.ac.uk/metagenomics/, PRJEB39025.

## Author Contributions

FG, AC, and SC: conceptualization. FG and AC: formal analysis, investigation, methodology, and validation. AC, SC, and MT: funding acquisition. AC: supervision. All authors: writing – original draft, review, and editing.

## Conflict of Interest

The authors declare that the research was conducted in the absence of any commercial or financial relationships that could be construed as a potential conflict of interest.

## Footnotes

1 https://docs.qiime2.org/2017.8/

2 https://github.com/qiime2/q2-demux

3 https://github.com/qiime2/q2-diversity

4 https://www.arb-silva.de/documentation/release-138/

5 https://github.com/qiime2/q2-feature-classifier

6 https://github.com/qiime2/q2-taxa
