# Supplementary material for: Effects of Zinc Pollution and Compost Amendment on the Root Microbiome of a Metal Tolerant Poplar Clone
Source: Front Microbiol. 2020 Jul 15;11:1677. doi: 10.3389/fmicb.2020.01677 (PMC7373765; doi:10.3389/fmicb.2020.01677)
Supplement: Supplementary file 1 [file Table_1.docx]

|  |  |  |
| --- | --- | --- |
| **Soil parameter** | **CNT** | CMP |
| pH | 7.33 ± 0.26 | 7.42 ± 0.03 |
| Organic Carbon (g kg^-1^) | 15.97 ± 0.53 | 24.73 ± 1.30 |
| Organic matter content (g kg^-1^) | 27.54 | 42.64 |
| CEC (cmol kg^-1^) | 0.39 ± 0.02 | 1.58 ± 0.01 |
| Total Carbon (%) | 3.0 ± 0.1 | 4.2 ± 0.1 |
| Total Nitrogen (%) | 0.14 ± 0.01 | 0.17 ± 0.01 |
| C/N | 21.4 | 24.7 |
| Zn | 134.49 ± 10.91 | 135.62 ± 14.88 |
| Cd | 0.74 ± 0.05 | 0.75 ± 0.08 |

Table SM1 Physical and chemical features of soils
