# Supplementary material for: Effects of Zinc Pollution and Compost Amendment on the Root Microbiome of a Metal Tolerant Poplar Clone
Source: Front Microbiol. 2020 Jul 15;11:1677. doi: 10.3389/fmicb.2020.01677 (PMC7373765; doi:10.3389/fmicb.2020.01677)
Supplement: Supplementary file 3 [file Data_Sheet_2.pdf]

Alpha rarefaction

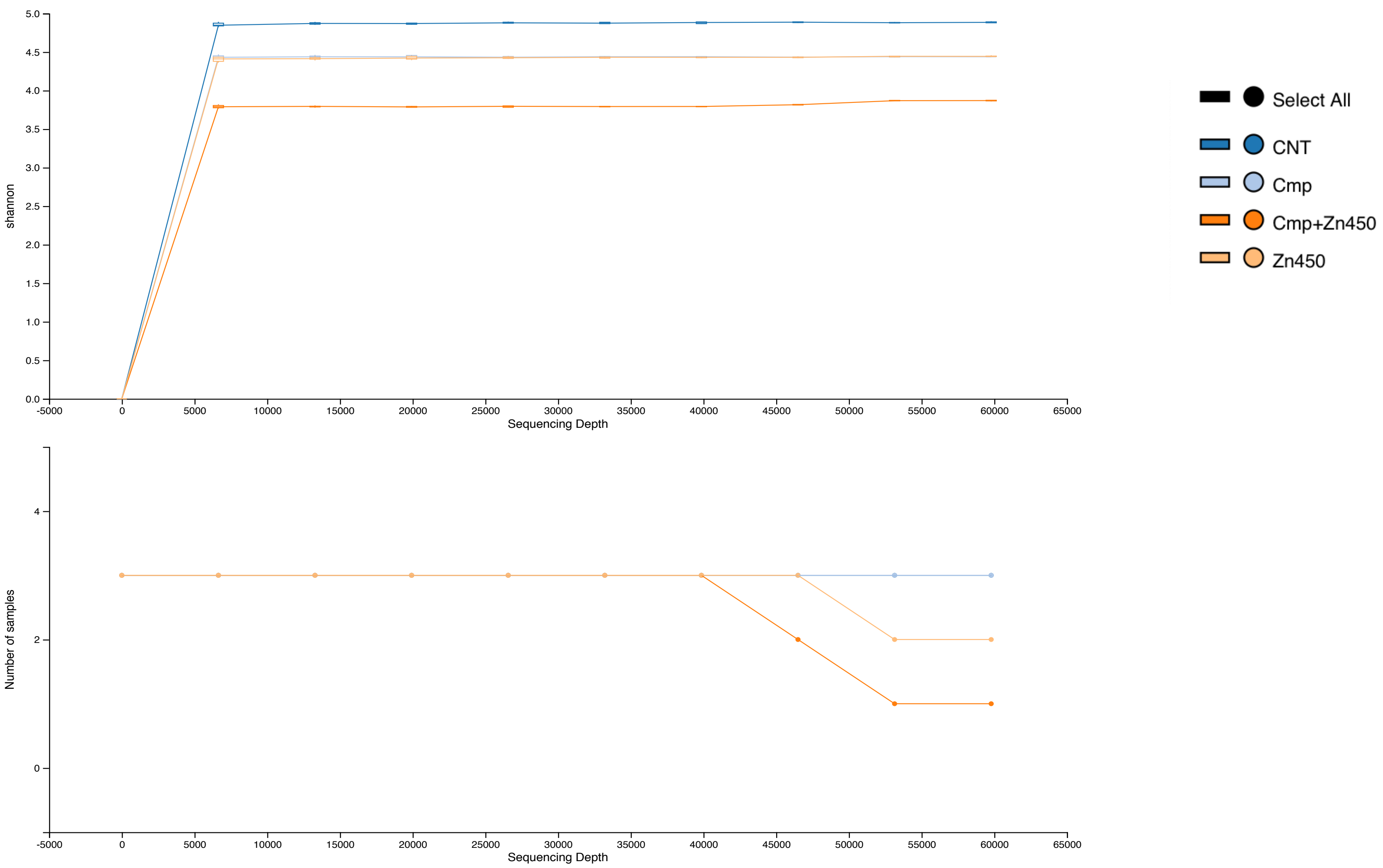

Fig. S2. Rarefaction curves and α-diversity plot of each sample group related to the bacterial diversity.
