# Supplementary material for: Effects of Zinc Pollution and Compost Amendment on the Root Microbiome of a Metal Tolerant Poplar Clone
Source: Front Microbiol. 2020 Jul 15;11:1677. doi: 10.3389/fmicb.2020.01677 (PMC7373765; doi:10.3389/fmicb.2020.01677)
Supplement: Supplementary file 4 [file Data_Sheet_3.pdf]

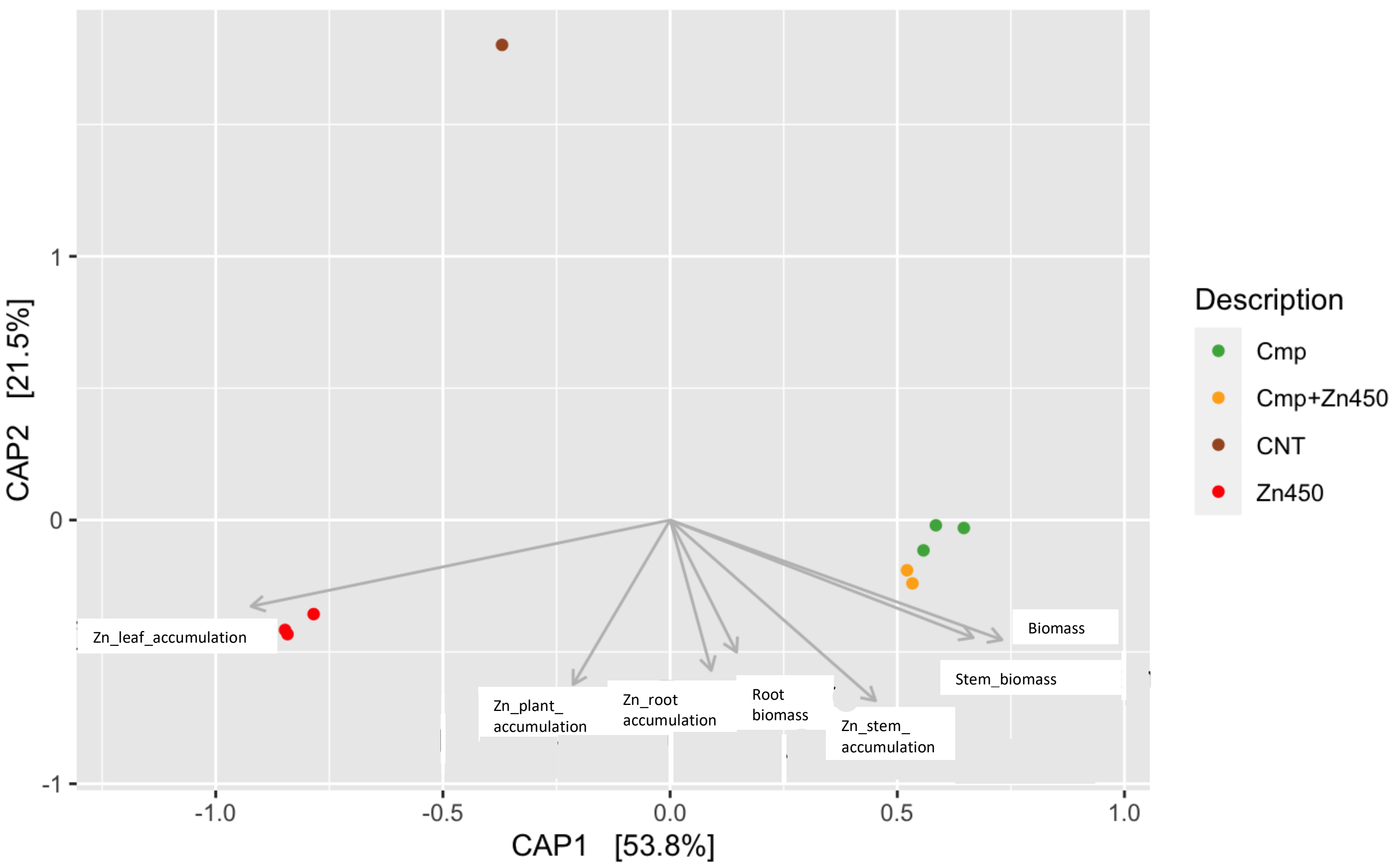

Fig. S3. Canonical analysis of principal coordinate (CAP) including the Bray Curtis distance of bacterial community. The axes created by a combination of: total biomass produced by plants (Biomass) and for each organ (Root_biomass, Stem_biomass, Leaf_biomass); Zn accumulation in plants and in each organ (Zn_plant_accumulation, Zn_root_accumulation, Zn_stem_accumulation, Zn_leaf_accumulation).
